# *trans*-1,2-Bis(3,5-dimethoxy­phen­yl)ethene

**DOI:** 10.1107/S160053680903116X

**Published:** 2009-08-15

**Authors:** Stefanie Ritter, Jörg-M. Neudörfl, Janna Velder, Hans-Günther Schmalz

**Affiliations:** a Department für Chemie der Universität zu Köln, Greinstrasse 4, 50939 Köln, Germany

## Abstract

The title compound, C_18_H_20_O_4_, was prepared in high yield from 3,5-dimethoxy­styrene *via* a Ru-catalysed homo-olefin metathesis. Exclusive formation of the *E*-configurated isomer was observed. Inter­estingly, one symmetric unit contains two mol­ecules adopting an *s*-*syn*-*anti* and and an all-*s*-*anti* conformation.

## Related literature

For the preparation of differently substituted stilbenes using a Ru-catalysed metathesis strategy, see: Velder *et al.* (2006 ▶). Alternative methodologies for the synthesis of ­oxy-function­alized stilbenes using Wittig-type olefinations or Heck couplings have been described by Kim *et al.* (2002 ▶), Lion *et al.* (2005 ▶), Botella & Nayera (2004 ▶) and Reetz *et al.* (1998 ▶). For the bioactivity of various stilbenes with a focus on their anti­cancer activity, see: Aggarwal *et al.* (2004 ▶); Wolter & Stein (2002 ▶); Fremont (2000 ▶); Jang *et al.* (1997 ▶); Wieder *et al.* (2001 ▶). For related structures and syntheses see: Yin *et al.* (2002 ▶); Uda *et al.* (2002 ▶).
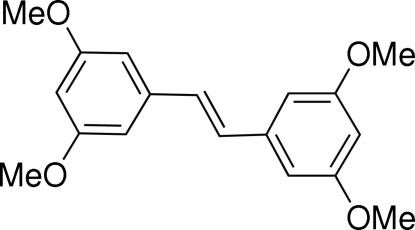


## Experimental

### 

#### Crystal data

C_18_H_20_O_4_
*M* *_r_* = 300.34
Monoclinic, 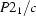

*a* = 7.1954 (3) Å
*b* = 9.4203 (4) Å
*c* = 22.6762 (5) Å
β = 93.783 (2)°
*V* = 1533.71 (10) Å^3^
*Z* = 4
Mo *K*α radiation
μ = 0.09 mm^−1^
*T* = 100 K
0.4 × 0.2 × 0.2 mm

#### Data collection

Nonius KappaCCD diffractometer
Absorption correction: none
7687 measured reflections
3320 independent reflections
2142 reflections with *I* > 2σ(*I*)
*R*_int_ = 0.041

#### Refinement

*R*[*F* ^2^ > 2σ(*F* ^2^)] = 0.055
*wR*(*F* ^2^) = 0.150
*S* = 1.03
3320 reflections
207 parameters
H-atom parameters constrained
Δρ_max_ = 0.24 e Å^−3^
Δρ_min_ = −0.24 e Å^−3^

### 

Data collection: *COLLECT* (Hooft, 1998 ▶); cell refinement: *DENZO* (Otwinowski & Minor, 1997 ▶); data reduction: *DENZO*; program(s) used to solve structure: *SHELXS97* (Sheldrick, 2008 ▶); program(s) used to refine structure: *SHELXL97* (Sheldrick, 2008 ▶); molecular graphics: *SCHAKAL99* (Keller 1999 ▶); software used to prepare material for publication: *PLATON* (Spek, 2009 ▶) and *enCIFer* (Allen *et al*., 2004 ▶).

## Supplementary Material

Crystal structure: contains datablocks global, I. DOI: 10.1107/S160053680903116X/hg2547sup1.cif

Structure factors: contains datablocks I. DOI: 10.1107/S160053680903116X/hg2547Isup2.hkl

Additional supplementary materials:  crystallographic information; 3D view; checkCIF report

## Figures and Tables

**Fig. 1.**
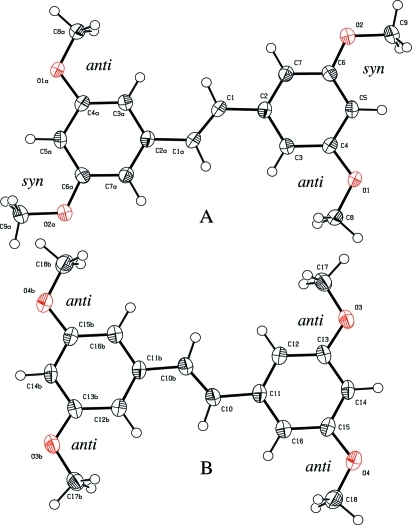
A view of (1). Displacement ellipsoids are drawn at the 50% probability level.

**Fig. 2.**
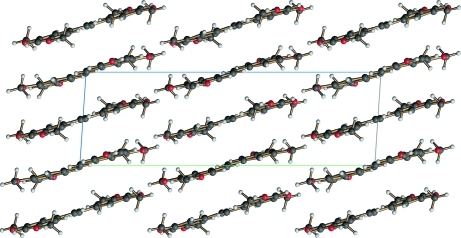
View of the unit cell along the b axis.

**Fig. 3.**
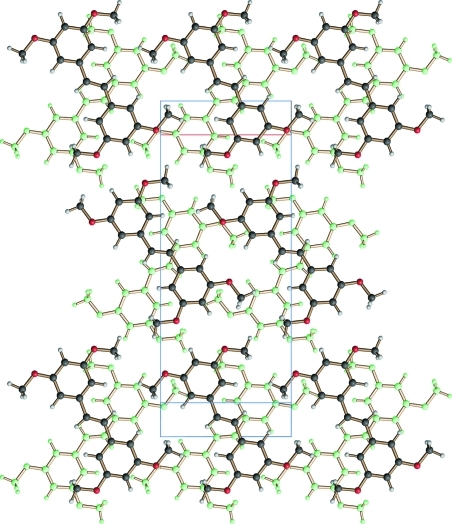
Top view of two pseudo layers.

## supplementary crystallographic information

### Comment

In recent years, polyhydroxylated stilbenes such as resveratrol have gained a tremendous importance especially due to their potential for the prevention and therapy of cancer (Aggarwal *et al.* (2004), Wolter *et al.* (2002), Fremont (2000), Jang *et al.* (1997)). In the course of our own research in the field of bioactive stilbenes (Wieder *et al.* (2001)) we were able to develop a highly efficient synthetic route towards symmetrically as well as unsymmetrically substituted E-stilbenes applying a Ru-catalyzed metathesis strategy (Velder *et al.* (2006)). Alternative strategies for the synthesis of stilbenes are based on Wittig-type olefinations or Heck couplings (Kim *et al.* (2002), Lion *et al.* (2005), Botella *et al.* (2004), Reetz *et al.* (1998). One of the compounds prepared was the title compound *trans*-1,2-bis-(3,5-dimethoxyphenyl)ethene. The asymmetric unit contains two molecules, A and B, both of which exhibit a center of symmetry (figure 1). The 3,5-dimethoxy groups of molecule B all adopt a s-anti configuration, whereas in molecule A, a s-*syn* as well as a s-anti conformation is found on both sides. Zhang and co-workers reported the same observation on a related structure (Yin *et al.*, 2002). The torsion angles between the benzene ring planes in molecule A are 0.2 (3)° (C1a—C1—C2—C3), which gives the molecule a planar shape. Molecule B, in contrast, adopts a slightly twisted conformation with a torsion angle of 7.0 (2)° (C10*b*—C10—C11—C12). The molecules form slightly twisted pseudo-layers which are arranged along the *b* axis (Fig. 2). In figure 3, two of those pseudo-layers are shown from the top view (with the front layer being displayed in dark and the retral layer in light green).

### Experimental

In a glove-box (Labmaster 130, mBraun), the catalyst (Grubbs-II, 2 mol %) was weighted into a 25 ml Schlenk tube, which was then sealed with a rubber septum. This was then taken out of the box, connected to an Ar-vacuum double manifold and equipped with a reflux condenser under argon. A solution of 3,5-dimethoxy-styrene (1 mmol) in CH_2_Cl_2_ (10 ml) was then added *via* syringe and the resulting solution was refluxed for 1.5 h under argon. After allowing the reaction mixture to cool to room temperature, the solvent was evaporated *in vacuo* and the crude product was purified by flash chromatography (SiO_2_, cyclohexane/ ethyl acetate = 10:1) to give 138 mg (0.46 mmol; 92%) of the homo metathesis product (1). mp. 142 °C (Uda *et al.*, 2002: 141–144 °C). 1H NMR (300 MHz, CDCl_3_): δ = 3.81 (s, 6H, OCH_3_), 6.4 (t, 1H, J = 2.1 Hz,H-4), 6.67 (d, 2H, J = 2.1 Hz, H-2, H-6), 7.00 (s, 1H, H-7); ^13^C NMR (75 MHz, CDCl_3_): δ = 55.4 (OCH_3_), 100.2 (C-4), 104.7 (C-2, C-6), 129.2 (C-7), 139.2 (C-1), 161.0 (C-3, C-5); HRMS, calcd for C_18_H_20_O_4_ (*M*^+^) 300.1361, found 300.136.

### Crystal data

**Table:**

C_18_H_20_O_4_	*F*(000) = 640
*M**_r_* = 300.34	*D*_x_ = 1.301 Mg m^−^^3^
Monoclinic, *P*2_1_/*c*	Melting point: 142 K
Hall symbol: -P 2ybc	Mo *K*α radiation, λ = 0.71073 Å
*a* = 7.1954 (3) Å	Cell parameters from 7687 reflections
*b* = 9.4203 (4) Å	θ = 2.3–27.0°
*c* = 22.6762 (5) Å	µ = 0.09 mm^−^^1^
β = 93.783 (2)°	*T* = 100 K
*V* = 1533.71 (10) Å^3^	Needle, colourless
*Z* = 4	0.4 × 0.2 × 0.2 mm

### Data collection

**Table:**

Nonius KappaCCD diffractometer	2142 reflections with *I* > 2σ(*I*)
Radiation source: fine-focus sealed tube	*R*_int_ = 0.041
graphite	θ_max_ = 27.0°, θ_min_ = 2.3°
φ and ω scans	*h* = −6→9
7687 measured reflections	*k* = −12→10
3320 independent reflections	*l* = −25→28

### Refinement

**Table:**

Refinement on *F*^2^	Primary atom site location: structure-invariant direct methods
Least-squares matrix: full	Secondary atom site location: difference Fourier map
*R*[*F*^2^ > 2σ(*F*^2^)] = 0.055	Hydrogen site location: inferred from neighbouring sites
*wR*(*F*^2^) = 0.150	H-atom parameters constrained
*S* = 1.03	*w* = 1/[σ^2^(*F*_o_^2^) + (0.0722*P*)^2^ + 0.274*P*] where *P* = (*F*_o_^2^ + 2*F*_c_^2^)/3
3320 reflections	(Δ/σ)_max_ < 0.001
207 parameters	Δρ_max_ = 0.24 e Å^−^^3^
0 restraints	Δρ_min_ = −0.24 e Å^−^^3^

### Special details

**Table:**

Geometry. All e.s.d.'s (except the e.s.d. in the dihedral angle between two l.s. planes) are estimated using the full covariance matrix. The cell e.s.d.'s are taken into account individually in the estimation of e.s.d.'s in distances, angles and torsion angles; correlations between e.s.d.'s in cell parameters are only used when they are defined by crystal symmetry. An approximate (isotropic) treatment of cell e.s.d.'s is used for estimating e.s.d.'s involving l.s. planes.
Refinement. Refinement of *F*^2^ against ALL reflections. The weighted *R*-factor *wR* and goodness of fit *S* are based on *F*^2^, conventional *R*-factors *R* are based on *F*, with *F* set to zero for negative *F*^2^. The threshold expression of *F*^2^ > σ(*F*^2^) is used only for calculating *R*-factors(gt) *etc*. and is not relevant to the choice of reflections for refinement. *R*-factors based on *F*^2^ are statistically about twice as large as those based on *F*, and *R*- factors based on ALL data will be even larger. The coordinates of the hydrogenatoms are constrained, and the U values of the H atoms are constrained relative to the *U*_eq_ of the atom the hydrogen binds to (1.2 for CH and CH_2_, 1.5 for CH_3_).

### Fractional atomic coordinates and isotropic or equivalent isotropic displacement parameters (Å^2^)

**Table:**

	*x*	*y*	*z*	*U*_iso_*/*U*_eq_	
O1	0.16550 (18)	0.11969 (13)	0.29039 (5)	0.0322 (4)	
O2	0.11990 (18)	0.52539 (13)	0.41414 (6)	0.0346 (4)	
C1	0.0096 (2)	0.07001 (18)	0.49846 (8)	0.0272 (4)	
H1	−0.0099	0.1220	0.5334	0.033*	
C2	0.0575 (2)	0.15286 (19)	0.44674 (8)	0.0256 (4)	
C3	0.0903 (2)	0.0879 (2)	0.39261 (8)	0.0268 (4)	
H3	0.0831	−0.0123	0.3885	0.032*	
C4	0.1329 (2)	0.17169 (19)	0.34549 (8)	0.0259 (4)	
C5	0.1450 (2)	0.31913 (19)	0.35017 (8)	0.0275 (4)	
H5	0.1748	0.3753	0.3173	0.033*	
C6	0.1123 (2)	0.38210 (19)	0.40394 (8)	0.0267 (4)	
C7	0.0691 (2)	0.2989 (2)	0.45200 (8)	0.0274 (4)	
H7	0.0475	0.3428	0.4886	0.033*	
C8	0.1666 (3)	−0.03060 (19)	0.28252 (8)	0.0312 (5)	
H8A	0.2575	−0.0734	0.3113	0.047*	
H8B	0.2006	−0.0530	0.2424	0.047*	
H8C	0.0424	−0.0687	0.2884	0.047*	
C9	0.1455 (3)	0.6156 (2)	0.36467 (9)	0.0359 (5)	
H9A	0.2645	0.5933	0.3482	0.054*	
H9B	0.1457	0.7149	0.3775	0.054*	
H9C	0.0437	0.6003	0.3344	0.054*	
O3	0.31579 (18)	0.49045 (14)	0.21349 (5)	0.0360 (4)	
O4	0.33384 (18)	0.03486 (14)	0.13362 (6)	0.0392 (4)	
C10	0.4952 (2)	0.43244 (19)	0.00794 (8)	0.0299 (4)	
H10	0.5284	0.3637	−0.0202	0.040 (6)*	
C11	0.4382 (2)	0.3787 (2)	0.06489 (8)	0.0273 (4)	
C12	0.4051 (2)	0.4693 (2)	0.11208 (8)	0.0289 (4)	
H12	0.4206	0.5689	0.1083	0.020 (5)*	
C13	0.3493 (2)	0.4114 (2)	0.16439 (8)	0.0288 (5)	
C14	0.3246 (2)	0.2665 (2)	0.17034 (8)	0.0303 (5)	
H14	0.2849	0.2285	0.2062	0.047 (6)*	
C15	0.3581 (2)	0.1774 (2)	0.12378 (8)	0.0296 (5)	
C16	0.4155 (2)	0.2328 (2)	0.07095 (8)	0.0293 (5)	
H16	0.4391	0.1711	0.0392	0.030 (5)*	
C17	0.3394 (3)	0.6410 (2)	0.20992 (9)	0.0373 (5)	
H17A	0.4679	0.6625	0.2011	0.056*	
H17B	0.3128	0.6845	0.2477	0.056*	
H17C	0.2536	0.6792	0.1785	0.056*	
C18	0.3671 (3)	−0.0593 (2)	0.08589 (9)	0.0386 (5)	
H18A	0.2853	−0.0340	0.0512	0.058*	
H18B	0.3415	−0.1571	0.0976	0.058*	
H18C	0.4974	−0.0514	0.0761	0.058*	

### Atomic displacement parameters (Å^2^)

**Table:**

	*U*^11^	*U*^22^	*U*^33^	*U*^12^	*U*^13^	*U*^23^
O1	0.0478 (8)	0.0283 (7)	0.0216 (7)	−0.0022 (6)	0.0118 (6)	0.0006 (6)
O2	0.0487 (8)	0.0268 (8)	0.0291 (8)	−0.0033 (6)	0.0080 (6)	0.0032 (6)
C1	0.0288 (9)	0.0332 (10)	0.0200 (9)	0.0004 (8)	0.0046 (7)	−0.0001 (8)
C2	0.0249 (9)	0.0291 (11)	0.0227 (10)	0.0000 (8)	0.0014 (7)	0.0040 (8)
C3	0.0280 (10)	0.0261 (10)	0.0265 (11)	−0.0010 (7)	0.0028 (8)	0.0025 (8)
C4	0.0256 (9)	0.0307 (11)	0.0216 (10)	0.0010 (8)	0.0034 (7)	0.0005 (8)
C5	0.0291 (10)	0.0288 (11)	0.0252 (10)	−0.0006 (8)	0.0053 (8)	0.0073 (8)
C6	0.0269 (10)	0.0249 (10)	0.0281 (11)	−0.0020 (8)	0.0008 (8)	0.0025 (8)
C7	0.0294 (10)	0.0295 (10)	0.0235 (10)	−0.0007 (8)	0.0040 (8)	0.0012 (8)
C8	0.0372 (11)	0.0303 (11)	0.0266 (11)	−0.0027 (8)	0.0056 (8)	−0.0018 (8)
C9	0.0444 (12)	0.0292 (11)	0.0342 (12)	−0.0031 (9)	0.0046 (9)	0.0078 (9)
O3	0.0443 (8)	0.0397 (8)	0.0251 (8)	−0.0033 (6)	0.0097 (6)	0.0002 (6)
O4	0.0489 (9)	0.0328 (8)	0.0370 (8)	−0.0008 (6)	0.0108 (7)	0.0093 (6)
C10	0.0324 (10)	0.0344 (10)	0.0232 (10)	−0.0013 (9)	0.0044 (8)	0.0020 (9)
C11	0.0248 (9)	0.0339 (11)	0.0234 (10)	−0.0013 (8)	0.0019 (7)	0.0060 (8)
C12	0.0285 (10)	0.0308 (11)	0.0275 (11)	−0.0019 (8)	0.0020 (8)	0.0048 (8)
C13	0.0252 (10)	0.0396 (12)	0.0218 (10)	−0.0017 (8)	0.0027 (7)	0.0028 (8)
C14	0.0294 (10)	0.0383 (12)	0.0237 (10)	−0.0005 (8)	0.0051 (8)	0.0093 (9)
C15	0.0266 (10)	0.0320 (11)	0.0304 (11)	−0.0018 (8)	0.0023 (8)	0.0090 (9)
C16	0.0282 (10)	0.0339 (12)	0.0259 (11)	−0.0001 (8)	0.0025 (8)	0.0018 (8)
C17	0.0434 (12)	0.0404 (12)	0.0287 (12)	−0.0045 (10)	0.0078 (9)	−0.0043 (9)
C18	0.0412 (12)	0.0330 (12)	0.0423 (13)	−0.0009 (9)	0.0070 (10)	0.0054 (10)

### Geometric parameters (Å, °)

**Table:**

O1—C4	1.376 (2)	O3—C13	1.374 (2)
O1—C8	1.427 (2)	O3—C17	1.432 (2)
O2—C6	1.370 (2)	O4—C15	1.375 (2)
O2—C9	1.429 (2)	O4—C18	1.432 (2)
C1—C1^i^	1.328 (3)	C10—C10^ii^	1.326 (4)
C1—C2	1.469 (2)	C10—C11	1.470 (2)
C1—H1	0.9500	C10—H10	0.9500
C2—C7	1.383 (3)	C11—C16	1.392 (3)
C2—C3	1.405 (2)	C11—C12	1.401 (3)
C3—C4	1.379 (2)	C12—C13	1.389 (2)
C3—H3	0.9500	C12—H12	0.9500
C4—C5	1.395 (3)	C13—C14	1.384 (3)
C5—C6	1.390 (2)	C14—C15	1.382 (3)
C5—H5	0.9500	C14—H14	0.9500
C6—C7	1.394 (2)	C15—C16	1.394 (2)
C7—H7	0.9500	C16—H16	0.9500
C8—H8A	0.9800	C17—H17A	0.9800
C8—H8B	0.9800	C17—H17B	0.9800
C8—H8C	0.9800	C17—H17C	0.9800
C9—H9A	0.9800	C18—H18A	0.9800
C9—H9B	0.9800	C18—H18B	0.9800
C9—H9C	0.9800	C18—H18C	0.9800
			
C4—O1—C8	117.99 (13)	C13—O3—C17	117.61 (14)
C6—O2—C9	117.31 (15)	C15—O4—C18	116.97 (14)
C1^i^—C1—C2	126.9 (2)	C10^ii^—C10—C11	126.3 (2)
C1^i^—C1—H1	116.6	C10^ii^—C10—H10	116.9
C2—C1—H1	116.6	C11—C10—H10	116.9
C7—C2—C3	119.73 (16)	C16—C11—C12	119.91 (16)
C7—C2—C1	118.41 (16)	C16—C11—C10	117.89 (17)
C3—C2—C1	121.87 (16)	C12—C11—C10	122.19 (17)
C4—C3—C2	119.10 (17)	C13—C12—C11	119.14 (17)
C4—C3—H3	120.5	C13—C12—H12	120.4
C2—C3—H3	120.5	C11—C12—H12	120.4
O1—C4—C3	124.02 (16)	O3—C13—C14	115.15 (16)
O1—C4—C5	114.20 (15)	O3—C13—C12	123.76 (17)
C3—C4—C5	121.77 (16)	C14—C13—C12	121.09 (17)
C6—C5—C4	118.58 (16)	C15—C14—C13	119.63 (16)
C6—C5—H5	120.7	C15—C14—H14	120.2
C4—C5—H5	120.7	C13—C14—H14	120.2
O2—C6—C5	124.17 (16)	O4—C15—C14	116.03 (16)
O2—C6—C7	115.49 (16)	O4—C15—C16	123.58 (17)
C5—C6—C7	120.34 (17)	C14—C15—C16	120.38 (17)
C2—C7—C6	120.47 (17)	C11—C16—C15	119.84 (17)
C2—C7—H7	119.8	C11—C16—H16	120.1
C6—C7—H7	119.8	C15—C16—H16	120.1
O1—C8—H8A	109.5	O3—C17—H17A	109.5
O1—C8—H8B	109.5	O3—C17—H17B	109.5
H8A—C8—H8B	109.5	H17A—C17—H17B	109.5
O1—C8—H8C	109.5	O3—C17—H17C	109.5
H8A—C8—H8C	109.5	H17A—C17—H17C	109.5
H8B—C8—H8C	109.5	H17B—C17—H17C	109.5
O2—C9—H9A	109.5	O4—C18—H18A	109.5
O2—C9—H9B	109.5	O4—C18—H18B	109.5
H9A—C9—H9B	109.5	H18A—C18—H18B	109.5
O2—C9—H9C	109.5	O4—C18—H18C	109.5
H9A—C9—H9C	109.5	H18A—C18—H18C	109.5
H9B—C9—H9C	109.5	H18B—C18—H18C	109.5
			
C1^i^—C1—C2—C7	179.3 (2)	C10^ii^—C10—C11—C16	172.5 (2)
C1^i^—C1—C2—C3	−0.3 (3)	C10^ii^—C10—C11—C12	−6.9 (4)
C7—C2—C3—C4	−0.2 (3)	C16—C11—C12—C13	−0.2 (3)
C1—C2—C3—C4	179.35 (16)	C10—C11—C12—C13	179.18 (17)
C8—O1—C4—C3	−4.1 (2)	C17—O3—C13—C14	179.90 (16)
C8—O1—C4—C5	176.49 (15)	C17—O3—C13—C12	0.3 (3)
C2—C3—C4—O1	−179.24 (16)	C11—C12—C13—O3	179.04 (16)
C2—C3—C4—C5	0.2 (3)	C11—C12—C13—C14	−0.5 (3)
O1—C4—C5—C6	179.31 (15)	O3—C13—C14—C15	−178.83 (15)
C3—C4—C5—C6	−0.2 (3)	C12—C13—C14—C15	0.8 (3)
C9—O2—C6—C5	6.2 (3)	C18—O4—C15—C14	179.78 (16)
C9—O2—C6—C7	−173.96 (15)	C18—O4—C15—C16	−1.1 (3)
C4—C5—C6—O2	179.99 (16)	C13—C14—C15—O4	178.80 (16)
C4—C5—C6—C7	0.2 (3)	C13—C14—C15—C16	−0.3 (3)
C3—C2—C7—C6	0.2 (3)	C12—C11—C16—C15	0.6 (3)
C1—C2—C7—C6	−179.33 (16)	C10—C11—C16—C15	−178.75 (16)
O2—C6—C7—C2	179.95 (15)	O4—C15—C16—C11	−179.43 (16)
C5—C6—C7—C2	−0.2 (3)	C14—C15—C16—C11	−0.4 (3)

Symmetry codes: (i) −*x*, −*y*, −*z*+1; (ii) −*x*+1, −*y*+1, −*z*.
